# Reduction of the forming voltage through tailored oxygen non-stoichiometry in tantalum oxide ReRAM devices

**DOI:** 10.1038/s41598-018-28992-9

**Published:** 2018-07-18

**Authors:** Katharina Skaja, Michael Andrä, Vikas Rana, Rainer Waser, Regina Dittmann, Christoph Baeumer

**Affiliations:** 1 Peter Grünberg Institute and JARA-FIT, FZ Jülich, D-52425 Jülich, Germany; 2 Institute for Electronic Materials, IWE2, RWTH Aachen University, D-52074 Aachen, Germany

## Abstract

In this study, we investigated the influence of oxygen non-stoichiometry on the resistive switching performance of tantalum oxide based memristive devices. Thin-films of tantalum oxide were deposited with varying sputter power and oxygen partial pressure. The electroforming voltage was found to decrease with increasing power density or decreased oxygen partial pressure, while the endurance remained stable and the resistance window *R*^*OFF*^/*R*^*ON*^ was found to increase. In-depth XPS analysis connects these observations to a controllable oxygen sub-stoichiometry in the sputter-deposited films. Our analysis shows that the decrease of the forming voltage results from an increase in carrier density in the as-prepared thin-films, which is induced by the presence of oxygen vacancies.

## Introduction

Memristive devices are an attractive emerging technology basis for nonvolatile memory (redox-based resistive switching random access memory, ReRAM)^1–4^ and for neuromorphic computing architectures^5,6^. The operation principle of these devices is based on a reversible resistance change of the dielectric layer in a simple capacitor structure. This resistance change is a result of voltage-driven migration of mobile donors, such as oxygen vacancies^3,4^. After a so-called electroforming step, which creates conductive oxygen vacancy channels (the “filaments”) in an insulating matrix within the dielectric layer^7–11^, the device resistance can be switched between a high resistance state (HRS) and a low resistance state (LRS).

The electroforming step is highly undesirable because of CMOS-incompatible high voltages and the time-consuming necessity of addressing every individual cell on a chip prior to operation. In addition, the stochastic nature of the electroforming process induces a significant device-to-device variation, i.e., the absolute resistance values of the LRS and HRS differ from cell to cell on the same chip^12^. This variation is due to fluctuations in filament size, shape, and location, and it is in fact often considered to be the biggest challenge that has to be overcome for a widespread implementation and use of ReRAM technology^13^.

Therefore, it is important to reduce the forming voltage. In the ideal case, the devices should be forming free, meaning that the forming voltage should fall into the same range as the switching voltages. In general, the forming voltage depends on the oxide thickness and initial resistance. Accordingly, the forming voltage can be reduced through a reduction of the active layer thickness^14,15^ and through an increase of the oxidizable metal thickness^14^. These methods, however, also lead to an undesirable increase of the leakage currents and, in turn, a decrease in the resistance window (*R*^*OFF*^/*R*^*ON*^) as well as a degradation of reliability and retention properties.

Following an alternative approach, Sharat *et al*. showed that amorphous, oxygen deficient HfO_2−*x*_ has a lower forming voltage than stoichiometric, polycrystalline HfO_2_^16,17^ and Kim *et al*. demonstrated that defects created by ion-bombardment lead to forming free Ta_2_O_5−*x*_-based devices^18^. In both cases, it was argued that oxygen deficiency may be responsible for the reduced forming voltage, but an unambiguous correlation between oxygen stoichiometry and forming voltage in the absence of additional structural differences was found only recently for the case of ReRAM devices based on tantalum oxide with a high percentage of suboxides^19^. In this study, Sharath *et al*. found a decrease in forming voltage for MBE-grown layers fabricated in oxygen-poor atmospheres, where all valence states of Ta including metallic Ta were observed. This, unfortunately, also led to a decrease of the resistance window for the case of forming-free devices. Understanding the effects enabling a reduction of the forming voltage is therefore mandatory to be able to maintain high resistance windows. At the same time, fabrication of such devices must be performed using wide-spread, inexpensive and CMOS-compatible processes, such as sputtering, to allow for integration into logic or memory arrays.

In this report, we therefore investigate the possibility to tailor the sub-stoichiometry of the oxide layer by a variation of the sputter deposition parameters to reduce the forming voltage of tantalum oxide-based ReRAM devices while preserving a high resistance window. To this end, we varied the O_2_ flow rate and the sputtering power during deposition of tantalum oxide thin-films, resulting in various degrees of comparably small oxygen deficiencies (≤10%). The electronic structure and the influence of the stoichiometry variation on the forming voltage and the resistance window were investigated by *in-situ* photoelectron spectscopy and *ex-situ* electrical characterization. We find that a decrease in the oxygen content leads to an increase of the electronic carrier density. This, in turn, causes a reduction of the forming voltage. For the small sub-stoichiometries investigated here, the resistance window was found to remain high for all samples, suggesting that a sweet spot may be found in the trade-off between reduction of the forming voltage and decrease of the resistance window.

## Results

15 nm Ta_2_O_5−*x*_ thin-films were obtained through reactive DC-sputtering. The thin-films were deposited in a defined atmosphere of Ar and O_2_; the total pressure was set to 3.5 × 10^−2^ mbar. Power density and the Ar/O_2_ flow rates were varied to achieve different stoichiometries (Table 1). All Ta_2_O_5−*x*_ thin-films were X-ray amorphous. The samples were transferred *in-situ* under UHV conditions from the sputter chamber to an XPS analysis chamber, see methods for details.

**Table 1:** Sputtering parameters for Ta_2_O_5−*x*_ thin-films.

power variation		variation of O_2_ content	
power density	O_2_/Ar	power density	O_2_/Ar
0.30 W/cm^2^	25.0%	1.20 W/cm^2^	25.0%
1.20 W/cm^2^	25.0%	1.20 W/cm^2^	14.3%
3.00 W/cm^2^	25.0%	1.20 W/cm^2^	10.0%
4.81 W/cm^2^	25.0%		

**Figure 1:**
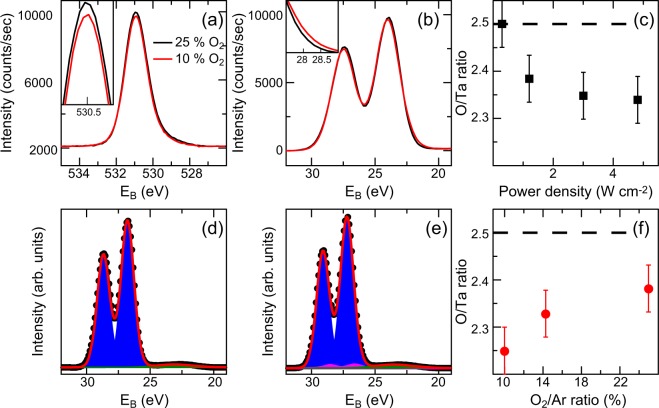
(**a**) Exemplary O 1s XPS spetrum for thin-films grown with a power density of 1.20 W/cm^2^ and a relative O_2_ flow rate of 25% (black line) and 10% (red line). Inset: zoom-in to the peak, showing the difference in intensity. (**b**) Ta 4f spectra for the same sample. In both cases, the spectra were shifted to the same binding energy for easier comparison and normalized to the Ta 4f intensity. Inset: zoom-in to the low binding energy edge of the Ta 4f spectrum, showing the differences in peak shape. (**c**) O/Ta ratio in dependence of the power density. (**d**) Fit of the Ta 4f spectrum for the thin-film deposited with 25% relative O_2_ flow rate. (**e**) Fit of the Ta 4f spectrum for the thin-film deposited with 10% relative O_2_ flow rate. Ta^5+^ (blue) and Ta^4+^ (pink) doublets as well as the O 2s level (green) were used for the fit (red line). (**f**) O/Ta ratio in dependence of the relative O_2_ flow rate.

Figure 1 summarizes the XPS investigation of the Ta_2_O_5−*x*_ thin-films. Representative O 1s and Ta 4f spectra for thin-films prepared with a power density of 1.20 W/cm^2^ and a relative O_2_ flow rate of 25% and 10% are shown in Fig. 1a and b. Slight differences in the O 1s peak height and Ta 4f peak shape can be detected. The reason for the differences in peak height becomes apparent from the determination of the elemental composition based on the integral intensity of the core level spectra shown in Fig. 1c and f. The relative O/Ta ratio varies with the preparation condition. Only the thin-film prepared with a comparably high O_2_ flow rate and a low power density corresponds to stoichiometric tantalum oxide, i.e. a O/Ta ratio of 2.5. All other samples exhibit varying degrees of oxygen deficiency. Intuitively, the oxygen content decreases with decreasing O_2_ flow rate^20^, but it also decreases with increasing power density. This behavior may be caused by an increased creation of oxygen vacancies through higher plasma energies (increase in UV-irradiation and particle-bombardment^21,22^). It may also be correlated to the deposition rate, which increases with the power density, resulting in a shorter time in which the samples stay at deposition conditions^23^.

To understand the shape of the Ta 4f spectra, we employed a peak model with several components. For the Ta 4f doublet, we fixed the area ratio to 0.75 and the spin orbit splitting to 1.87 eV^24,25^. The Ta 4f_7/2_ for Ta_2_O_5_ is located at a binding energy of 26.8 ± 0.4 eV^24^ and the O 2s level at 23 eV^26^ was included. For fairly oxidized thin-films, this peak model leads to satisfactory fit of the experimental data (Fig. 1e). For comparably reduced thin-films, an additional Ta 4f doublet shifted 0.6 eV towards lower binding had to be added to the peak model. This component represents the reduced species TaO_2_ and the observed energy shift is in good agreement with literature^25,27,28^. This component is only visible for the sample prepared with an O_2_/Ar flow ratio of 10%, with a relative intensity of about 2%; for all other samples it is below the detection limit. Here we would like to note that in contrast to the highly reduced thin-films investigated in ref.^19^, we do not observe Ta in a valence state lower than 4+.

**Figure 2:**
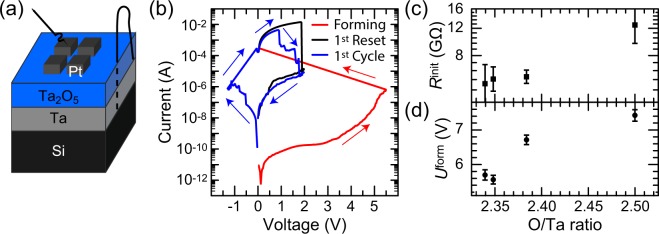
(**a**) Schematic illustration of the device layout. (**b**) *I*–*V* characteristics for a Ta_2_O_5_ cell prepared with a power density of 4.81 W/cm^2^ (O/Ta = 2.34). (**c**) Stoichiometry dependence of the initial resistance. (**d**) Stoichiometry dependence of the forming voltage. All values were averaged for at least 20 cells for each stoichiometry.

Having established a set of Ta_2_O_5−*x*_ thin-films with known off-stoichiometry, we now turn to the investigation of the influence of the stoichiometry on the forming and switching characteristics of ReRAM devices using four selected stoichiometries. To this end, we fabricated 50 nm Ta/15 nm Ta_2_O_5−*x*_/70 nm Pt devices. Quasistatic voltage cycles were applied to the top electrode, while the bottom electrode remained grounded. A schematic of the device and measurement setup is shown in Fig. 2a, further information is presented in the methods section.

Before the ReRAM devices were resistively switched, the initial resistance of the cells was measured. All samples are highly insulating and have an initial resistance in the range of several GΩ (cf. Fig. 2c), close to the detection limit of the measurement setup. We observe a general trend of decreasing initial resistance with decreasing oxygen content.

In order to switch the initially highly insulating cells, an electroforming step is necessary. Therefore, all cells were formed with a first voltage sweep to positive voltages. A current limit of 300 μA was used to prevent hard breakdown. Afterwards the cells can be switched in counter-eightwise polarity^29^. In Fig. 2a the *I-V*-characteristic is shown for a cell with a O/Ta ratio of 2.34. A forming step enables switching between LRS and HRS with a Set voltage ≈−1.0 V (i.e., the voltage at which the current limit is reached during the Set sweep) and a Reset current of ≈3.7 mA (i.e., the maximum current during the Reset sweep). As a function of stoichiometry, the forming voltages increase with increasing oxygen content, as illustrated in Fig. 2d. For O/Ta ≈ 2.5, we find a forming voltage of ≈7.4 V, which decreases to ≈5.7 V for O/Ta ≈ 2.34. The odd data point at O/Ta ≈ 2.35 lies within the same range. Note that the currents used here are comparably high because of the very large device size. For smaller devices, much lower switching currents can be achieved^30^.

**Figure 3:**
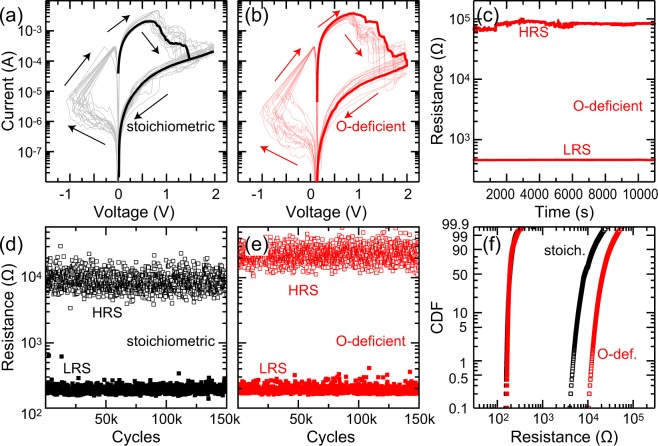
(**a**) *I*–*V* characteristics for a Ta_2_O_5_ cell prepared with a power density of 0.30 W/cm^2^ (O/Ta = 2.5). (**b**) *I*–*V* characteristics for a Ta_2_O_5−x_ cell prepared with a power density of 4.81 W/cm^2^ (O/Ta = 2.34). In both cases, ten sweeps from two representative devices are shown along with an average Reset loop, which was obtained by averaging more than 100 sweeps and nine devices for each stoichiometry. A total of 25 devices were measured for both stoichiometries. (**c**) Retention characteristic for a similar Ta_2_O_5−x_ cell prepared with a power density of 1.2 W/cm^2^ (O/Ta = 2.39). (**d**) Endurance measurement for a Ta_2_O_5_ cell prepared with a power density of 0.30 W/cm^2^ (O/Ta = 2.5). (**e**) Endurance measurement for a Ta_2_O_5−x_ cell prepared with a power density of 4.81 W/cm^2^ (O/Ta = 2.34). Every 100^th^ measurement point is shown. (**f**) Cumulative distribution function (CDF) of the measurements shown in panels (d) and (e).

Regarding the switching performance, we compared the extreme cases of almost stoichiometric Ta_2_O_5_ and the most oxygen deficient Ta_2_O_5−*x*_ in Fig. 3 (O/Ta ratios of 2.5 and 2.34). On first sight, the *I*–*V* characteristics appear similar for both devices (Fig. 3a and b), but closer inspection reveals that the resistance windows are different, with an *R*^*OFF*^/*R*^*ON*^ ratio of approximately 500 and 2000 for samples with O/Ta ratios of 2.5 and 2.34, respectively.

Further, we also characterized the retention and endurance behavior of our devices. The retention was found to be independent of the stoichiometry for the time scales investigated here, as shown for an exemplary device in Fig. 3c. Further characterization is beyond the scope of this work. The endurance was found to be sufficient for all stoichiometries investigated here. Exemplary endurance measurements for almost stoichiometric Ta_2_O_5_ and oxygen deficient Ta_2_O_5−*x*_ confirm the observation of increased memory window for the oxygen deficient devices, i.e., the sample with the lower forming voltage (Fig. 3d–f). This is contrary to the trend observed before, where a decreasing resistance window was observed for decreasing forming voltage^14,15,19^.

## Discussion

**Figure 4:**
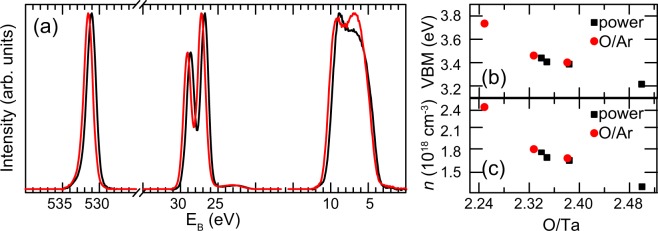
(**a**) Ta 4f, O 1s and VB spectra for thin-films grown with a power density of 1.20 W/cm^2^ and a relative O_2_ flow rate of 25% (black line) and 10% (red line). (**b**) VBM positions and (**c**) charge carrier concentrations for the different stoichiometries.

To understand the correlation between oxygen deficiency and forming voltage, we investigated the electronic structure of our samples in more detail through a more profound comparison of the XPS spectra. Figure 4a shows an overview of the different core level and valence band spectra for samples prepared with an O/Ta ratio of 2.24 and 2.38, respectively. The energy scale is referred to the Fermi level *E*_*F*_ of metallic tantalum. (Note that for the purpose of comparing the peak shape in Fig. 1, we had artificially shifted the spectra to identical peak positions, while we are now comparing their position on an absolute binding energy scale). For all spectra, a rigid shift towards higher binding energy is visible for the sample with lower oxygen content. This can be explained by a relative shift of the conduction band towards the Fermi level^31,32^, which is also correlated with an increased donor dopant concentration^33^.

In order to quantify the shift and the resulting carrier concentration differences, the energy shifts for the different thin-films was measured through the position of the valence band maximum (VBM), which was extracted from the zero-photoemission intensity intercept of a linear regression fit of the low-binding-energy edge of the valence band spectra^34,35^. An overview of all VBM fitting results is shown in Fig. 4b. With decreasing oxygen content, the VBM shifts monotonically towards higher binding energies.

Using this shift, we can determine the carrier density of the Ta_2_O_5−*x*_ thin-films from the position of the conduction band *E*_*C*_, which is given by

1 $${E}_{C}-{E}_{F}={E}_{F}-{E}_{{\rm{VBM}}}+{E}_{g}\mathrm{.}$$

Here, *E*_*g*_ is the energy of the band gap, which was reported to be 4.2 eV for amorphous tantalum oxide^36,37^. Possible reductions of the band gap with decreasing oxygen content^19^ are expected to be negligible for the small sub-stoichiometries investigated here. The electronic charge carrier concentration can then be expressed as^38^

2 $$n={N}_{C}\,\exp (-\frac{{E}_{C}-{E}_{F}}{{k}_{B}T})$$

with the effective density of states *N*_*C*_

3 $${N}_{C}=2{(\frac{2\pi {m}_{n}^{\ast }{k}_{B}T}{{h}^{2}})}^{\tfrac{3}{2}}\mathrm{.}$$

The effective mass ${m}_{n}^{\ast }$ was reported to be 0.3 ⋅ *m*_*e*_ for Ta_2_O_5−*x*_^39^. The calculated charge carrier concentrations as a function of the O/Ta ratio are shown in Fig. 4c. With decreasing oxygen content, the charge carrier concentration increases. This results in different amounts of *n-*type doping, thus indicating that the oxygen deficiency causes an increase in electrical conductivity, which is also consistent with the observed initial resistances.

During the forming step, a similar current of ≈2 μA is reached for all stoichiometries before a sudden jump to the current limit occurs. This characteristic forming current is reached at lower voltages for oxygen-deficient devices. Therefore, Joule-heating-activated ionic motion may be enabled at lower voltages for oxygen deficient samples, thus contributing to the reduced forming voltage. At the same time, the power during forming was found to increase gently from 1.6 mW to 2.1 mW with increasing oxygen content. This shows that the absolute amount of Joule heating was actually higher for stoichiometric samples. It thus appears likely that in addition to higher conductivity and resulting higher amount of Joule heating for a given voltage, an additional mechanism contributes to the decrease in forming voltage. Possible scenarios include stronger confinement for sub-stoichiometric films (i), changes of the local electric field at the interfaces (ii) or a more direct contribution from the oxygen vacancies already present in the film (iii). In scenario (i), the confinement results in smaller filament diameters and, accordingly, higher current densities. For case (ii), one might argue that the electric field enhancement at the interfaces could be a function of the stoichiometry, as the width of space charge zones strongly depends on the number of dopants. For scenario (iii) we follow the mechanisms proposed by Sharma *et al*., who suggested that during the pre-stage of electroforming, Poole-Frenkel or hopping-based mechanisms contribute to the sudden conductivity increase and confinement of the current transport to a single filament^40^. As these mechanisms strongly depend on the number of defects mediating the electronic transport, and therefore the number of oxygen vacancies, the non-stoichiometric films may show enhanced current filamentation and voltage non-linearity. These, in turn, accelerate the forming process for a given power. While the experimental proof for one of these mechanisms (or a combination thereof) has to remain subject of future research, they are in line with the observed increase in oxygen-vacancy-induced conductivity observed here.

The reason for surprising observation of enhanced memory window with decreasing forming voltage lies in the leakage current of our devices: As we are using rather subtle sub-stoichiometries (in contrast to previous reports using very high oxygen non-stoichiometry), the leakage current for the oxygen-deficient films is still much smaller than the HRS current, which appears to be limited by the filament resistance. Our reduced forming voltage, in turn, induces a lower current overshoot during the forming process, resulting in a higher resistance of the filament itself, and thus to an improved resistance window^41^. This is most likely a special case for our comparably low sub-stoichiometries, indicating that in the trade-off between forming voltage and resistance window, a sweet spot may be found through stoichiometry optimization for specific applications.

In conclusion, our analysis of memristive devices based on Ta_2_O_5−*x*_ thin-films shows that the reduction in forming voltage is caused by a decrease in the oxygen concentration, which can be controlled through appropriate choice of the deposition parameters. The oxygen vacancies lead to a relative shift of the conduction band towards the Fermi level, which results in an increase in carrier density in the as-deposited oxide, as is apparent from the XPS analysis and electrical characteristics. Our results therefore yield the desired clear correlation between the oxygen defect structure and the device properties and may thus contribute to the achievement of forming-free devices while maintaining high resistance windows.

## Methods

The sputter-deposition rates were determined by X-ray reflectometry and scale linearly with the sputtering power density. The samples were transferred *in-situ* under UHV conditions from the sputter chamber to a PHI 5000 Versa Probe II XPS analysis chamber with AL K_*α*_ X-ray irradiation with a spot size of ≈100 μm. For all thin-films a survey scan and single core level spectra were taken at normal emission with a pass energy of 23.5 eV and under electron neutralization. No significant carbon signal nor other contaminants could be detected, confirming appropriate preparation and measurement conditions. The data were quantified and analyzed by using *CasaXPS* version 2.3.16 using Shirley background subtraction. The sensitivity factors (RSF) were calibrated to stoichiometric tantalum oxide powder. Error bars are calculated based on the reasonable assumption of a 1% precision.

Layer stacks consisting of a 50 nm tantalum bottom electrode and 15 nm Ta_2_O_5−*x*_ active layer were deposited by reactive DC-sputtering. The total pressure was set to 3.5 × 10^−2^ mbar, the oxygen partial pressure was adjusted through a variation of the Ar/O_2_ flow rates. A detailed discussion of the impact of the Ar/O_2_ flow rate on the plasma kinetics is given by^42^. This was followed by *ex-situ* deposition of Pt electrodes by electron-beam evaporation through a shadow mask. The top electrodes have a thickness of 70 nm and the pads have a size of 50 × 50 μm. Each wafer had 1700 individual cells. The electrical contact to the bottom electrode was enabled via wire bonding to a chip carrier; the top electrode was contacted with tungsten whisker probes. Quasistatic voltage cycles were applied to the top electrode using a *Keithley 2611* SourceMeter^32^, while the bottom electrode remained grounded.

Before the ReRAM devices were resistively switched, the initial resistance of the cells was measured with an *I-V*-sweep from −0.5 V to 0.5 V, voltages sufficiently small so that they do not change the device electrically. The initial resistance values were extracted at a voltage of 60 mV.

For the endurance measurement, the resistance was measured at a voltage of 100 mV after a Set operation with 300 μA and a Reset operation with 1.5 V or 2 V, with a pulse length of 0.2 ms. The difference between stoichiometric and oxygen deficient samples was consistent for all Reset voltages tested here. For the retention measurement, the current was measured at a voltage of 100 mV.

### Data availability

The datasets generated during and/or analysed during the current study are available from the corresponding author on reasonable request.

## References

[1] Zhirnov VV (2010). Memory devices: Energy-space-time tradeoffs. Proc. IEEE.

[2] Chen A (2013). A comprehensive crossbar array model with solutions for line resistance and nonlinear device characteristics. IEEE Trans. Electron Devices.

[3] Waser R, Aono M (2007). Nanoionics-based resistive switching memories. Nat. Mater..

[4] Waser R, Dittmann R, Staikov G, Szot K (2009). Redox-based resistive switching memories - nanoionic mechanisms, prospects, and challenges. Adv. Mater..

[5] Indiveri, G., Linn, E. & Ambrogio, S. Reram-based neuromorphic computing. In Ielmini, D. & Waser, R. (eds.) *Resistive Switching: From Fundamentals of Nanoionic Redox Processes to Memristive Device Applications* (Wiley, 2016).

[6] Prezioso M, Bayat FM, Hoskins B, Likharev K, Strukov D (2016). Self-adaptive spike-time-dependent plasticity of metal-oxide memristors. Scientific Reports.

[7] Strachan JP (2010). Direct identification of the conducting channels in a functioning memristive device. Adv. Mater..

[8] Baeumer C (2015). Spectromicroscopic insights for rational design of redox-based memristive devices. Nat. Commun..

[9] Celano U (2015). Imaging the three-dimensional conductive channel in filamentary-based oxide resistive switching memory. Nano Letters.

[10] Buckwell M, Montesi L, Hudziak S, Mehonic A, Kenyon A (2015). J. Conductance tomography of conductive filaments in intrinsic silicon-rich silica rram. Nanoscale.

[11] Brivio S, Tallarida G, Cianci E, Spiga S (2014). Formation and disruption of conductive filaments in a hfo2/tin structure. Nanotechnology.

[12] Wei, Z. *et al*. Highly reliable taox reram and direct evidence of redox reaction mechanism. *IEEE Tech. Dig*. (2008).

[13] Baeumer C (2017). Subfilamentary networks cause cycle-to-cycle variability in memristive devices. ACS Nano.

[14] Govoreanu, B. *et al*. 10 × 10 nm^2^ hf/hfo_*x*_ crossbar resistive ram with excellent performance, reliability and low-energy operation. In *IEEE International Electron Devices Meeting*, 31.6.1–31.6.4 (2011).

[15] Kim, W. *et al*. Lowering forming voltage and forming-free behavior of ta2o5 reram devices. In *Proceedings of the 43th European Solid-State Device Research Conference (ESSDERC), Lausanne, Switzerland, September 12–15, 2016*, 164–167 (Proceedings of the 43th European Solid-State Device Research Conference(ESSDERC), Lausanne, Switzerland, September 12–15, 2016, 2016).

[16] Sharath SU (2014). Towards forming-free resistive switching in oxygen engineered hfo2-x. Appl. Phys. Lett..

[17] Sharath SU (2014). Thickness independent reduced forming voltage in oxygen engineered hfo2 based resistive switching memories. Appl. Phys. Lett..

[18] Kim, W. *et al*. Forming-free metal-oxide reram by oxygen ion implantation process. In *2016 IEEE International Electron Devices Meeting (IEDM), San Francisco, USA, December 3–7, 2016* (2016 IEEE International Electron Devices Meeting (IEDM), San Francisco, USA, December 3–7, 2016, 2016).

[19] Sharath SU (2016). Impact of oxygen stoichiometry on electroforming and multiple switching modes in tin/taox/pt based reram. Appl. Phys. Lett..

[20] Goldfarb I (2012). Electronic structure and transport measurements of amorphous transition-metal oxides: observation of fermi glass behavior. Appl. Phys. A Mater. Sci. Process..

[21] Zywitzki, O. *et al*. Structure and properties of crystalline titanium oxide layers deposited by reactive pulse magnetron sputtering. *Surface and Coatings Technology* **180–181**, 538–543, (2004). Proceedings of Symposium G on Protective Coatings and Thin Films-03, of the E-MRS 2003 Spring Conference.

[22] Cormier P-A (2014). Titanium oxide thin film growth by magnetron sputtering: Total energy flux and its relationship with the phase constitution. Surface and Coatings Technology.

[23] Tachikawa T (2012). Metal-to-insulator transition in anatase tio2 thin films induced by growth rate modulation. Appl. Phys. Lett..

[24] Sanz J, Hofmann S (1983). Auger electron spectroscopy and x-ray photoelectron spectroscopy studies of the oxidation of polycristalline tantalum an niobium at room temperature and low oxygen pressures. Journal of Less-Common Metals.

[25] Kerrec O, Devilliers D, Groult H, Marcus P (1998). Study of dry and electrogenerated ta2o5 and ta/ta2o5/pt structures by xps. Materials Science and Engineering. B.

[26] Moulder, J., Stickle, W., Sobol, P. & Bomben, K. *Handbook of X Ray Photoelectron Spectroscopy* (Physical Electronics, 1995).

[27] Diaz B (2012). Chromium and tantalum oxide nanocoatings prepared by filtered cathodic arc deposition for corrosion protection of carbon steel. Surf. Coat. Technol..

[28] Diaz B (2013). Tantalum oxide nanocoatings prepared by atomic layer and filtered cathodic arc deposition for corrosion protection of steel: Comparative surface and electrochemical analysis. Electrochimica Acta.

[29] Cooper D (2017). Anomalous resistance hysteresis in oxide reram: Oxygen evolution and reincorporation revealed by *in situ* tem. Adv. Mater..

[30] Tsai C-L, Xiong F, Pop E, Shim M (2013). Resistive random access memory enabled by carbon nanotube crossbar electrodes. ACS Nano.

[31] Andrae M (2017). Oxygen partial pressure dependence of surface space charge formation in donor-dopedsrtio3. APL Mater..

[32] Skaja K (2015). Avalanche-discharge-induced electrical forming in tantalum oxide-based metal-insulator-metal structures. Advanced Functional Materials.

[33] Bondi RJ, Desjarlais MP, Thompson AP, Brennecka GL, Marinella MJ (2013). Electrical conductivity in oxygen-deficient phases of tantalum pentoxide from first-principles calculations. J. Appl. Phys..

[34] Chambers SA, Droubay T, Kaspar TC, Gutowski M (2004). Experimental determination of valence band maxima forsrtio3, tio2, and sro and the associated valence band offsets with si(001). J. Vac. Sci. Technol. B.

[35] Schafranek R, Li S, Chen F, Wu W, Klein A (2011). Pbtio_3_/srtio_3_ interface: Energy band alignment and its relation to the limits of fermi level variation. Phys. Rev. B.

[36] Shvets V (2008). Electronic structure and charge transport properties of amorphous ta2o5 films. Journal of Non-Crystalline Solids.

[37] Rico V (2009). Wetting angles on illuminated ta2o5 thin films with controlled nanostructure. The Journal of Physical Chemistry C.

[38] Sze, S. M. & Ng, K. K. *Physics of Semiconductor Devices* 3 edn (Wiley, 2007).

[39] Houssa M (2000). Trap-assisted tunneling in high permittivity gate dielectric stacks. J. Appl. Phys..

[40] Sharma A, Noman M, Abdelmoula M, Skowronski M, Bain J (2014). Electronic instabilities leading to electroformation of binary metal oxide-based resistive switches. Adv. Funct. Mater..

[41] Gilmer, D. C. *et al*. Effects of rram stack configuration on forming voltage and current overshoot. In *2011 3rd IEEE International Memory Workshop (IMW)*, 1–4 (2011).

[42] Trennepohl W, Bretagne J, Gousset G, Pagnon D, Touzeau M (1996). Modelling of an reactive magnetron discharge used for deposition of chromium oxide. Plasma Sources Sci. Technol..

